# Identification of Multiple *PlOSCs* Involved in the Biosynthesis Pathway of Triterpenoids in *Paeonia lactiflora*

**DOI:** 10.3390/ijms27104410

**Published:** 2026-05-15

**Authors:** Yufeng Zhao, Juan Guo, Jiyu Zhang, Jian Wang, Luqi Huang

**Affiliations:** 1 College of Animal Science and Technology, Tarim University, Alar 843300, China; zhaoyf20@lzu.edu.cn; 2 The China Academy of Chinese Medical Sciences, No. 16 Dongzhimen Neinan Street, Dongcheng District, Beijing 100091, China; guojuanzy@163.com (J.G.); jianwang2021@126.com (J.W.); 3 State Key Laboratory of Grassland Agro-Ecosystems, Key Laboratory of Grassland Livestock Industry Innovation, Ministry of Agriculture and Rural Affairs, College of Pastoral Agriculture Science and Technology, Lanzhou University, Lanzhou 730020, China

**Keywords:** triterpenoids, 2,3-oxidosqualene cyclases, *Paeonia lactiflora*, β-amyrin

## Abstract

Triterpenoid saponins are important bioactive compounds synthesized through the isoprenoid pathway, in which 2,3-oxidosqualene serves as a precursor of triterpenoid saponins. In this study, we identified and characterized eight oxidosqualene cyclase (*PlOSC*) genes in *Paeonia lactiflora* using molecular cloning and bioinformatic analyses. Full-length cDNAs of *PlOSCs* (*PlOSC1*–*PlOSC8*) were cloned, and the protein sequences exhibited significant similarities to known cyclases, including β-amyrin synthase and cycloartenol synthase. Phylogenetic analysis revealed distinct groups of *PlOSCs* corresponding to lupeol, β-amyrin, and cycloartenol synthases. Sequence alignment confirmed the presence of highly conserved motifs, including the “SDCTAE” and “QW” motifs, which are crucial for cyclization and stability in *PlOSCs.* To determine the functional roles of *PlOSCs*, we conducted functional expression studies in *Saccharomyces cerevisiae*. The results showed that *PlOSC3* and *PlOSC6* are monofunctional β-amyrin synthases that produce β-amyrin in yeast culture, as confirmed through GC-MS analysis. Further investigation of *PlOSC* gene expression in various tissues indicated that *PlOSC3* was predominantly expressed in roots, whereas *PlOSC6* was highly expressed in leaves. Corresponding metabolite analyses revealed that triterpenoid accumulation was significantly higher in roots than in leaves, suggesting tissue-specific biosynthesis and accumulation patterns in triterpenoid biosynthesis. These findings contribute to our understanding of the regulation of triterpenoid biosynthesis in *P. lactiflora* and provide insights into the functional roles of OSCs in triterpenoid and nortriterpenoid formation.

## 1. Introduction

*Paeonia lactiflora*, commonly known as Chinese peony, belongs to the genus Paeonia [1]. It is an economically important plant that can be used for both medicinal and ornamental purposes [2] and has been widely used as a traditional Chinese medicine with a long history in China, North Korea, Japan, Mongolia, and Russia to relieve pain, relieve spasms, remove blood stasis, and promote menstrual flow [3,4,5]. Terpenoids are structurally diverse natural products that can be obtained from plants, microorganisms, and marine animals [6,7]. According to the number of isoprene units that make up the terpenoid skeleton, terpenoids can be divided into hemiterpenoids, monoterpenoids, sesquiterpenoids, diterpenoids, disesquiterpenoids, triterpenoids, and polyterpenoids [8,9]. Natural terpenoids are widely used in various fields such as medicine, food, and chemical engineering; for example, artemisinin is the most effective antimalarial drug, while paclitaxel and ginsenosides can be used as antitumor drugs [10,11,12].

Triterpenoids are the most structurally complex class of secondary metabolites in plants. These compounds are widely distributed in plants and possess diverse physiological and ecological functions, such as regulating plant growth and development and defending against pathogen attacks [13]. Simultaneously, triterpenoids hold significant application prospects in the pharmaceutical field owing to their broad spectrum of pharmacological activities [14]. In terms of plant growth and development, triterpenoids participate in regulating cell division, elongation, and tissue differentiation. Phytosterols, an important class of triterpenoids, regulate the fluidity and stability of cell membranes and play a crucial role in plant hormone signal transduction [15]. In recent years, research has found that the oxidized products of triterpenoids, such as ursolic acid and oleanolic acid, can promote plant growth and participate in stress responses [16]. Triterpenoids perform various roles in the physiological and biochemical processes of plants, including regulating growth and development, enhancing stress resistance, participating in plant–microbe interactions, and defending against pests and diseases [17,18].

Oxidosqualene cyclases (OSCs) are key enzymes that catalyze the biosynthesis of triterpenes. OSCs primarily function by converting 2,3-oxidosqualene into various triterpenes [19]. In recent years, researchers have successfully cloned and functionally characterized multiple OSC genes in model plants, such as *Arabidopsis thaliana* and *Solanum lycopersicum* (tomato). For example, in Arabidopsis, *AtOSC1* catalyzes the production of cycloartenol, a critical intermediate in the plant sterol biosynthesis pathway [20]. Furthermore, studies on *SlOSC* genes in tomatoes have demonstrated that the triterpenes they catalyze play significant roles in plant disease resistance and defense mechanisms [21].

Among the triterpenoids identified, oleanolic acid and ursolic acid have notable biological activities. Oleanolic acid is commonly used in clinical applications and is synthesized from the β-amyrin skeleton. OSC enzymes play a pivotal role in determining the structural diversity of triterpenoids by guiding 2,3-oxidosqualene toward different skeleton types through cyclization. However, triterpenoid skeleton synthase activity has not been reported in *P. lactiflora*. In this study, we cloned eight full-length *PlOSC* cDNAs from *P. lactiflora* and characterized their functions in yeast, establishing a foundation for further triterpenoid biosynthesis research in this species.

## 2. Results

### 2.1. Molecular Cloning of Full-Length cDNA Encoding PlOSCs

Eight annotated genes encoding 2,3-oxidosqualene cyclases were selected and named *PlOSC1*–*PlOSC8* (Table S1). The six full-length coding sequences (CDS) of eight *PlOSCs* were amplified by means of PCR with cDNA as a template, and the length of the encoded proteins of *PlOSCs* was between 786 and 791 amino acids (information on *PlOSCs* is presented in Table S1).

**Figure 1:**
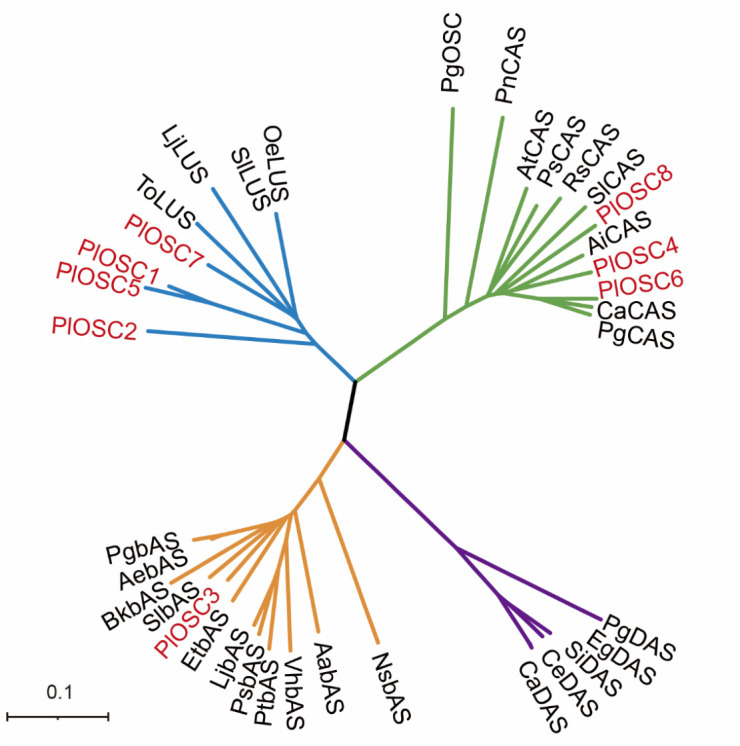
A phylogenetic tree of a wide range of 2,3-oxidosqualene cyclase amino acid sequences was constructed using the neighbor-joining method. Sequences were selected from GenBank based on their authentication in the literature (unless otherwise indicated). Information on the selected sequences is presented in Table S4. The four major clades are color-coded based on enzyme function: blue (lupeol synthase, LUS), green (cycloartenol synthase, CAS), orange (β-amyrin synthase, β-AS), and purple (dammarenediol synthase, DAS). PlOSC proteins identified in *Paeonia lactiflora* are highlighted in red. The scale bar indicates the genetic distance (0.1 substitutions per site).

BLAST results (Table S1) showed that the amino acid sequences of *PlOSC1*, *PlOSC2*, and *PlOSC5* shared 78.12%, 75.93%, and 75.30% sequence identity with *CfLUS*, *OeLUS*, and *EgLUS*, respectively (*Cornus florida*, *Olea europaea*, and *Eucalyptus grandis*; lupeol synthase, XP_059636756, CAA3029550, and XP_039166480, respectively). *PlOSC4*, *PlOSC6*, and *PlOSC8* shared 87.05%, 85.34%, and 84.58% sequence identity with *PpCAS*, *CfCAS*, and *PaCAS*, respectively (*Prunus persica*, *C. florida,* and *Populus alba*; cycloartenol Synthase, XP_007225240, XP_0596345, and KAJ6915670). *PlOSC3* exhibited 89.45% identity with *CpbAS* (*Cyclocarya paliurus* β-amyrin synthase WNA08415). *PlOSC7* exhibited 84.30% identity with *Lagerstroemia speciosa oxidosqualene cyclase (LsOSC*, AZS32327). In the phylogenetic tree of *PlOSCs* (Figure 1), *PlOSC3* is closely related to β-amyrin synthase from *Solanum lycopersicum* (tomato) and clusters together with three other β-amyrin synthases derived from *Aralia elata*, *Panax ginseng*, and *Bupleurum kaoi*. *PlOSC4*, *PlOSC6*, and *PlOSC8* clustered with other cycloartenol synthases in the same branch. The BLAST and phylogenetic results were consistent with the annotations of the *P. lactiflora* genome database, and three types of oxidosqualene cyclases were identified in *P. lactiflora*.

**Figure 2:**
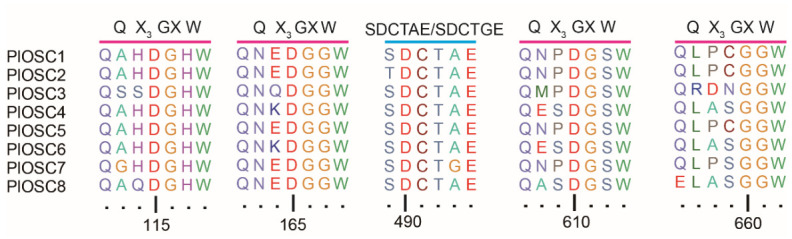
Multiple sequence alignment of 2,3-oxidosqualene cyclase (OSC) predicted amino acid sequences from *P. lactiflora*. Conserved domains critical for OSC activity are highlighted: the repeated Q X_3_GXW motifs are indicated by magenta lines, and the catalytic SDCTAE/SDCTGE motif is indicated by a cyan line.

Multiple sequence alignments revealed that all *PlOSCs* contained a highly conserved “SDCTAE motif” (except *PlOSC7* containing the “SDCTGE motif” and *PlOSC2* containing the “TDCTAE motif”) in the same position homologous to the highly conserved “DxDD motif” in 2,3-oxidosqualene cyclases [22,23], which is involved in the polycyclization reaction of 2,3-oxidosqualene (Figure 2). Additionally, all *PlOSCs* contained repeats of the QW motif, which is important for stabilizing the structure of OSCs and carbocation intermediates [24].

### 2.2. Functional Identification of PlOSCs in Yeast

**Figure 3:**
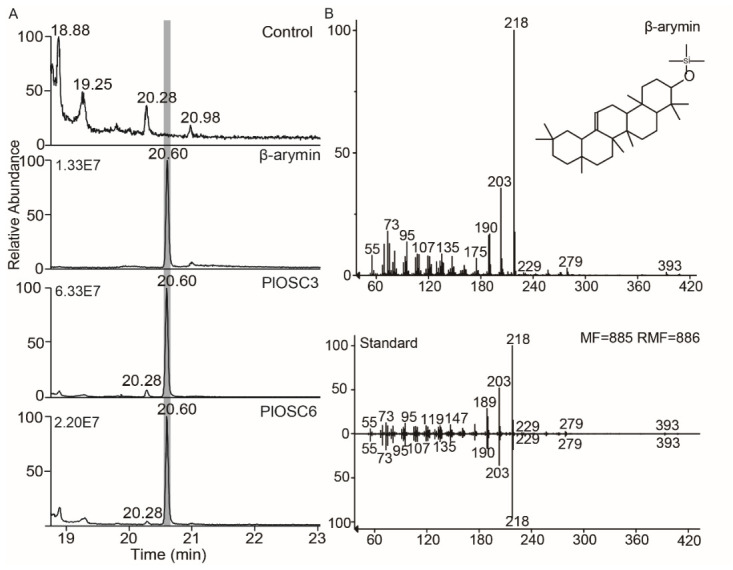
GC/MS analysis of yeast extracts (in GL77) expressing heterologous *PlOSCs*. (**A**) Total ion chromatograms showing the enzymatic reaction products of PlOSC3 and PlOSC6, along with a negative control (empty vector) and a β-amyrin reference standard. The gray-shaded region indicates the retention time corresponding to authentic β-amyrin (20.60 min), which is consistently observed in the standard and enzyme reaction samples but absent in the control. (**B**) Mass fragmentation patterns of the target peak from PlOSC3 reaction products, which are identical to those of the β-amyrin standard, confirming the product identity.

2,3-Oxidosqualene cyclase (OSC) catalyzes the key step in the cyclization of 2,3-oxidosqualene to the skeletons of triterpenoids and sterols. OSCs are responsible for the main scaffold cyclization of triterpenes and sterols. To characterize the biofunction of the putative *PlOSCs*, the full-length sequences of the eight *PlOSCs* were cloned into the pESC-Trp vector and transformed into an ERG7-deficient yeast strain. The extracts of cells expressing *PlOSC3* and *PlOSC6* were analyzed by means of GC-MS, and only a peak with the same mass spectral characteristics and retention time as the β-amyrin standard was observed, with no corresponding peak detected in the vector control (Figure 3). Thus, the results clearly demonstrated that *PlOSC3* and *PlOSC6* were monofunctional β-amyrin synthases.

### 2.3. Preliminary Prediction of Major Formation of Triterpenoid Skeletons

**Figure 4:**
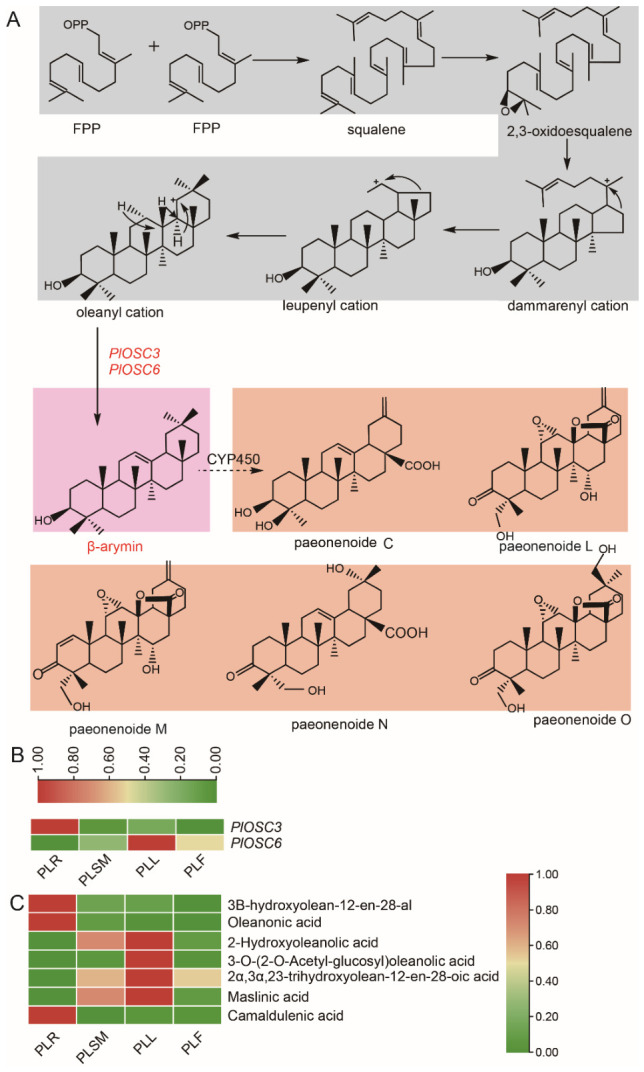
Proposed mechanism of major triterpene skeleton formation and heatmap of selected *PlOSC* gene expression levels. (**A**) Proposed mechanism of major triterpene skeleton formation. Squalene is synthesized through a condensation reaction of farnesyl diphosphate (FPP). 2,3-Oxidosqualene is cyclized by β-amyrin synthase to form the triterpene β-amyrin (indicated in gray). β-amyrin (indicated in pink) may be further converted into triterpenes or degraded triterpenes (indicated in orange) in peony through the catalytic action of *CYP450* enzymes. (**B**) Tissue-specific expression patterns of β-amyrin synthase genes *PlOSC3* and *PlOSC6* in *Paeonia lactiflora*. (**C**) Tissue-specific accumulation of triterpenoids in *Paeonia lactiflora*. Heatmap of *PlOSC* gene expression levels, with red indicating high expression and green indicating low expression.

The biosynthesis of triterpenoid saponins begins with the isoprenoid pathway, in which farnesyl pyrophosphate (FPP) undergoes cyclization to produce 2,3-oxidosqualene. Two FPP molecules were connected in a head-to-tail manner to form squalene, a 30-carbon product. This reaction is catalyzed by squalene synthase (SS). Squalene is subsequently oxidized by squalene epoxidase (SE) to 2,3-oxidosqualene, marking the initial step toward cyclization, which ultimately produces triterpenoid saponins. Further structural modifications, such as oxidation, substitution, and glycosylation, are mediated by various enzymes, resulting in the formation of diverse skeletons [25]. Pentacyclic carbon frameworks are derived from the 2,3-oxidosqualene precursor through a series of processes catalyzed by oxidosqualene cyclases (OSCs; Figure 4A). These processes yield triterpenoid skeletons, including oleanane (β-amyrin), ursane (α-amyrin), betulin, and damaran.

In this study, a heatmap analysis of the expression levels of *PlOSC* genes involved in the biosynthesis of triterpenoid skeletons revealed distinct tissue-specific patterns. Notably, *PlOSC3* was highly expressed in the roots, whereas *PlOSC6* was predominantly expressed in the leaves (Figure 4B). These findings suggest that the accumulation of triterpenoid compounds may vary among different plant tissues, potentially correlating with the tissue-specific roles of these compounds in plant development and stress responses. Further analysis revealed that the accumulation of triterpenoid compounds was significantly higher in the roots than in the leaves. This is likely linked to the high expression levels of *PlOSC3* in the roots, suggesting that the roots may be the primary site for triterpenoid biosynthesis and accumulation. In contrast, although *PlOSC6* was highly expressed in the leaves, the accumulation of triterpenoid compounds in the leaves was relatively low(Figure 4C). This discrepancy may be due to the influence of other regulatory factors or the specific metabolic demands of the leaves for other secondary metabolites.

## 3. Discussion

Functional characterization of oxidosqualene cyclases (OSCs) is pivotal for deciphering the biosynthetic pathways of structurally diverse triterpenoids in medicinal plants. In this study, we successfully cloned and functionally identified eight OSC genes from *Paeonia lactiflora*, among which *PlOSC3* and *PlOSC6* were characterized as monofunctional β-amyrin synthases. This finding not only expands the repository of functional OSCs in plants but also provides direct genetic evidence for the biosynthesis of oleanane-type triterpenoids, a major class of bioactive components in *P. lactiflora* [26]. It is well known that α-amyrin serves as the skeletal structure of ursane-type triterpenoids. Based on our current findings, the screened genes may include enzymes capable of converting 2,3-oxidosqualene into α-amyrin. This may necessitate expanding the scope of gene screening or increasing the diversity of experimental methods and chassis strains [27,28].

A significant finding of our work is the distinct tissue-specific expression patterns of functional β-amyrin synthases. *PlOSC3* was highly expressed in the roots, correlating with the significantly higher accumulation of total triterpenoids in this tissue. In contrast, *PlOSC6* is predominantly expressed in the leaves, where triterpenoid accumulation is lower. This pattern suggests sophisticated spatial regulation of triterpenoid biosynthesis in *P. lactiflora*. Roots, as the primary medicinal parts, appear to be the major sites for the production and storage of oleanane-type triterpenoid precursors. The high expression of *PlOSC3* likely channels the metabolic flux towards β-amyrin in roots. The role of *PlOSC6* in leaves remains intriguing; its product (β-amyrin) may serve as a substrate for the synthesis of leaf-specific derivatives or may be involved in local defense responses, with lower accumulation possibly due to rapid turnover or further modification. This tissue-specific divergence mirrors the findings in other plants, where OSCs and downstream pathways are compartmentalized to fulfill organ-specific physiological roles [29].

To date, 30 triterpenoid compounds have been discovered in plants of the Paeonia genus, including 11 nortriterpenoids, reported between 2011 and 2024. Research on the biosynthesis of demethylated noroleanane triterpenoids, both domestically and internationally, is still in its infancy, with only 30 demethylated triterpenoids containing a C20 (29) double bond reported [30]. This also indicates that the genus Paeonia is suitable for studying nortriterpenoid biosynthesis pathways. Research on the biosynthesis of various demethylated noroleanane triterpenoids is still in its infancy. Our identification of functional β-amyrin synthases (*PlOSC3/6*) provides the essential first committed step towards the biosynthesis of noroleanane-type compounds in *P. lactiflora*. Subsequent transformations are likely mediated by cytochrome P450 enzymes (CYPs), which are known to catalyze site-specific oxidations, ring rearrangements, and possibly demethylation [31]. Transcriptomic studies on *P. lactiflora* have identified numerous CYP genes, with some showing co-expression with OSC genes or high expression in roots. Developing and clarifying their research results will help in the rapid enrichment and preparation of highly active target compounds through artificial cultivation or synthesis, contributing to the rational utilization and protection of these natural resources.

In the future, our research group will focus on β-amymin and screen for potential genes with high homology to reported genes, such as *CYP450*, that have demethylation and other effects. We plan to further study the triterpenoids and pharmacological activities of the oleananes reported in *P. lactiflora*.

## 4. Materials and Methods

### 4.1. Plant Materials 

The *P. lactiflora* plant material used in this study (all two years old) was grown in the same public planting base in Heze, Shandong Province. Plants were obtained in June 2021. Three tissues from each plant were examined during the *P. lactiflora* flowering period, including the roots, stems, flowers, and leaves. All collected tissues were quickly frozen in liquid nitrogen and stored at −80 °C before RNA extraction.

### 4.2. Total RNA Isolation and cDNA Synthesis

Total RNA was isolated from *P. lactiflora* leaves using an RNA isolation kit (HuaYueYang Biotechnology, Beijing, China). RNA was reverse-transcribed into first-generation cDNA using the PrimerScript™ RT Reagent Kit with a gDNA eraser (TaKaRa Corp., Dalian, China).

### 4.3. Isolation and Cloning of PlOSC Coding Sequences

To identify OSC genes in *P. lactiflora*, the resulting unique genes were annotated using a combination of Nr, Nt, Pfam, KOG/COG, Swiss-Prot, KEGG, and GO. According to transcriptome annotation results, gene-specific primers (Tables S2 and S3) were designed to isolate *PlOSCs* based on RNA-seq data. The PCR products were purified and cloned into a T-vector (pEASY-Blunt Zero Simple Vector, TransGen, Beijing, China). The recombinant plasmids were isolated using a kit and sequenced by Sangon Biotech (Shanghai) Co., Ltd. (Shanghai, China).

### 4.4. Bioinformatics Analyses of PlOSCs

We used the SMART BLAST tool at the NCBI (National Library of Medicine, Bethesda, MD, USA, http://www.ncbi.nlm.nih.gov/ (accessed on 10 May 2025) NCBI BLAST+ version 2.17.0) to search the database for sequence comparison and analyzed the nucleotide and deduced amino acid sequences. Clustal W software (Clustal W version 2.0, Japan) was used to implement multiple sequences. Phylogenetic analysis was performed using MEGA 7.0 software with the neighbor-joining method. Confidence values for individual branches were measured from 1000 bootstrap replicates of the original sequence data points.

### 4.5. Functional Characterization of PlOSCs

The multiple cloning sites of the vector and the ORFs of *PlOSCs* were amplified using specific primers. Amplicons were digested with BamHI restriction enzyme (New England Biolab, Ipswich, MA, USA) and inserted into the expression vector, pESC-Trp, with a pEASY-Uni Seamless Cloning and Assembly Kit (TransGen, Beijing, China). The confirmed recombinant plasmids were extracted and introduced into the ERG7-deficient yeast mutant (*Saccharomyces cerevisiae*, ATCC, cell line number: 4021900, -ERG7, -Trp) using the Frozen-EZ Yeast Transformation II™ Kit (Zymo Research, Irvine, CA, USA) according to the technical manual.

SD-Trp media (synthetic dextrose minimal medium without tryptophan, containing 20 g·L^−1^ glucose) was used to select the yeast harboring the expression vector and grown at 30 °C for 72 h. A single colony was selected and inoculated into SD-Trp medium at 30 °C for 72 h. An expanded culture was inoculated (1:100) into 100 mL SD-Trp (containing 20 g·L^−1^ glucose) under the same conditions for 72 h (to OD_600_ = 2–3). The cells were collected by means of centrifugation at 3500 rpm for 8 min, resuspended in induction SD-Trp media (containing 20 g·L^−1^ galactose), and grown at 30 °C for 72 h.

An equal volume of ethyl acetate was used to extract the products after cell disruption (three times). The extracts were dried with N_2_ and re-dissolved in 150 μL ethyl acetate for GC–MS analysis. GC–MS analysis was performed using a Trace1310 system coupled with a TSQ 8000 mass selective detector (Thermo Scientific, San Jose, CA, USA). The GC-MS detection procedure was as follows: the initial column oven temperature was set at 50 °C and held for 2 min, then increased to 210 °C at a rate of 30 °C/min, followed by a further increase to 250 °C at 5 °C/min, and finally ramped to 280 °C at 40 °C/min and held for 5 min. The ion source temperature was set to 280 °C. The final products were identified by comparing the retention times and mass spectral data with those of known enzymatic products or authentic standards. Finally, the results were verified through searches and confirmation using a built-in database.

### 4.6. Metabolomic Analysis

A non-targeted metabolomic approach was employed to analyze the volatile compounds from different tissues of *P*. *lactiflora*. A total of 12 samples were collected, comprising four distinct tissue types with three biological replicates per tissue. Sample extraction was performed using headspace solid-phase microextraction (HS-SPME) technology by Wuhan Metware Biotechnology Co., Ltd., Wuhan, China. The extracted volatile compounds were subsequently analyzed using a gas chromatography–mass spectrometry (GC-MS) system equipped with an Agilent 8890 GC instrument. Identification of volatile compounds was achieved by comparing their mass spectra with those in the MWGC and NIST mass spectral libraries, thereby constructing a comprehensive compound database.

For terpenoid analysis, samples were subjected to freeze-drying prior to extraction at Wuhan Metware Biotechnology Co., Ltd. The extracted terpenoid compounds were analyzed using an ultra-performance liquid chromatography–electrospray ionization–tandem mass spectrometry (UPLC-ESI-MS/MS) system, consisting of a Shimadzu Nexera X2 UPLC system coupled with an Applied Biosystems 4500 Q TRAP mass spectrometer. Identification of terpenoid compounds was achieved by comparing their mass spectra with those in the MWGC mass spectral library, thereby constructing a dedicated terpenoid database.

The relative abundance of terpenoid and volatile secondary metabolites across different tissue types was visualized using heatmaps. To investigate the relative content changes of metabolites among different groups, the relative abundances of all differentially accumulated metabolites (DAMs) identified from pairwise group comparisons were subjected to Z-score normalization. Subsequently, K-means clustering analysis was performed to classify the metabolites into distinct clusters based on their accumulation patterns across tissue types.

For gene expression analysis, heatmaps were generated using the TBtools software version 2.115. The colors in the heatmap represent normalized relative expression values rather than raw expression values (e.g., FPKM) or fold change values. During the normalization process, the expression data were subjected to min–max normalization according to the following formula:

$$x^{\prime }=\frac{x-min(x)}{max(x)-min(x)}$$

where x represents the expression value of a given gene across different samples, and $x^{\prime }$ is the normalized value. After normalization, all gene expression values were scaled to a range of [0, 1], enabling the heatmap colors to intuitively display the expression trends of each gene across different tissue types.

## 5. Conclusions

In this study, we successfully identified and characterized eight oxidosqualene cyclase (*PlOSC*) genes in *P. lactiflora*. Through molecular cloning, phylogenetic analysis, and functional expression in yeast, we demonstrated that *PlOSC3* and *PlOSC6* encode monofunctional β-amyrin synthases that are involved in the biosynthesis of oleanane-type triterpenoids. The tissue-specific expression patterns of these genes, with *PlOSC3* predominantly expressed in roots and *PlOSC6* in leaves, correlated with the higher accumulation of triterpenoid compounds in root tissues. These findings provide crucial genetic insights into the biosynthesis of triterpenoid skeletons in *P. lactiflora* and establish a foundational framework for future investigations of the complex pathways leading to the formation of bioactive nortriterpenoids in this medicinally important species.

## Data Availability

The original contributions presented in this study are included in the article/Supplementary Material. Further inquiries can be directed to the corresponding authors.

## Supplementary Materials

The following supporting information can be downloaded at https://www.mdpi.com/article/10.3390/ijms27104410/s1.
