# Supplementary material for: Identification of Multiple PlOSCs Involved in the Biosynthesis Pathway of Triterpenoids in Paeonia lactiflora
Source: Int J Mol Sci. 2026 May 15;27(10):4410. doi: 10.3390/ijms27104410 (PMC13207941; doi:10.3390/ijms27104410)
Supplement: Supplementary file 1 [file ijms-27-04410-s001.zip › ijms-4208152-supplementary.pdf]

Identification multiple *PlOSCs* involved in the biosynthesis pathway of triterpenoids in *Paeonia lactiflora*

Zhao Yufeng[1,2], Guo Juan[2] , Zhang Jiyu[3*], Wang Jian[2]

1. College of Animal Science and Technology, Tarim University, Alar 843300, China; 1933142587@qq. com;
2. The China Academy of Chinese Medical Sciences No. 16 Dongzhimen Neinan Street, Dongcheng District, Beijing 100091, China. guojuanzy@163.com;
3. State Key Laboratory of Grassland Agro-ecosystems, Key Laboratory of Grassland Livestock Industry Innovation, Ministry of Agriculture and Rural Affairs, College of Pastoral Agriculture Science and Technology, Lanzhou University, Lanzhou 730020, China; zhangjy@lzu.edu.cn;
4. *Correspondence: zhangjy@lzu.edu.cn;

*** Correspondence:**

Jiyu Zhang

Address: State Key Laboratory of Grassland Agro-ecosystems, Key Laboratory of Grassland Livestock Industry Innovation, Ministry of Agriculture and Rural Affairs, College of Pastoral Agriculture Science and Technology, Lanzhou University, Lanzhou 730020, China;

E-mail: zhangjy@lzu.edu.cn

Table S1 RAN-seq data of *PlOSCs*

ID	Number of AA	BlastP result	BlastP percent(%)	BlastP species	Gene bank NO.
PlOSC1	787	lupeol synthase	78.12%	*Cornus florida*	XP_059636756
PlOSC2	786	lupeol synthase	75.93%	*Olea europaea subsp. Europaea*	CAA3029550
PlOSC3	789	beta-amyrin synthase	89.45%	*Cyclocarya paliurus*	WNA08415
PlOSC4	787	cycloartenol synthase	87.05%	*Prunus persica*	XP_007225240
PlOSC5	787	lupeol synthase	75.30%	*Eucalyptus grandis*	XP_039166480
PlOSC6	787	cycloartenol synthase	85.34%	*Cornus florida*	XP_059634521
PlOSC7	783	oxidosqualene cyclase	84.30%	*Lagerstroemia speciosa*	AZS32327
PlOSC8	791	cycloartenol synthase	84.58%	*Populus alba x Populus x berolinensis*	KAJ6915670

Table S2 Primers for cloning the ORFs of PlOSCs

**Names**	**Sequence (5`→3`)**
PlOSC1	F: ATGTGGAAGCTAAAG<br>R:TTATGCAAACAAAAC
PlOSC2	F: ATGTGGAAGCTGAAGATAG<br>R:TTATGCACGCACAACTC
PlOSC3	F: ATGTGGAAGCTGAAGATAGCAGAAG<br>R:TTAGCAAGGCAATGGAACTCG
PlOSC4	F: ATGTGGAAGCTGAAGATC<br>R:TCAGGAGACTTGCAATAC
PlOSC5	F: ATGTGGAAGCTAAAGATAG<br>R:TTATCCAATCAGAACATGTC
PlOSC6	F: ATGTGGAAGCTGAAGATCG<br>R:TCAGGAGACTTGCAATACC
PlOSC7	F: ATGTGGAAGCTCAAGATAG<br>R:TCATGCAAACTGAACGC
PlOSC8	F: ATGTGGAAGCTCAAGATCGGTGGTG<br>R:TCAGAGGGCTAGGAGCACCTGAGAGCGG

Table S3 Primers for vector construction of *PlOSCs*

**Names**	**Sequence (5`→3`)**
PlOSC1	bamh1-F: acgtcaaggagaaaaaaccccggatccATGTGGAAGCTAAAG<br>bamh1-R:tagtgagtcgtattacggatccTTATGCAAACAAAAC
PlOSC2	bamh1-F: acgtcaaggagaaaaaaccccggatccATGTGGAAGCTGAAGATAG<br>bamh1-R:tagtgagtcgtattacggatccTTATGCACGCACAACTC
PlOSC3	bamh1-F: acgtcaaggagaaaaaaccccggatccATGTGGAAGCTGAAGATAGCAGAAG<br>bamh1-R:tagtgagtcgtattacggatccTTAGCAAGGCAATGGAACTCG
PlOSC4	bamh1-F: acgtcaaggagaaaaaaccccggatccATGTGGAAGCTGAAGATC<br>bamh1-R:tagtgagtcgtattacggatccTCAGGAGACTTGCAATAC
PlOSC5	bamh1-F: acgtcaaggagaaaaaaccccggatccATGTGGAAGCTAAAGATAG<br>bamh1-R:tagtgagtcgtattacggatccTTATCCAATCAGAACATGTC
PlOSC6	bamh1-F: acgtcaaggagaaaaaaccccggatccATGTGGAAGCTGAAGATCG<br>bamh1-R:tagtgagtcgtattacggatccTCAGGAGACTTGCAATACC
PlOSC7	bamh1-F: acgtcaaggagaaaaaaccccggatccATGTGGAAGCTCAAGATAG<br>bamh1-R:tagtgagtcgtattacggatccTCATGCAAACTGAACGC
PlOSC8	bamh1-F: acgtcaaggagaaaaaaccccggatccATGTGGAAGCTCAAGATCGGTGGTG<br>bamh1-R:tagtgagtcgtattacggatccTCAGAGGGCTAGGAGCACCTGAGAGCGG

Table S4 The informtion of other OSCs

Gene name	Organism Name	
VhbAS	Vaccaria hispanica	DQ915167
PtbAS	*Polygala tenuifolia*	EF107623
PsbAS	*Pisum sativum*	AB034802
PgbAS	*Panax ginseng*	AB009030
NsbAS	*Nigella Sativa*	FJ013228
LjbAS	*Lotus japonicus*	BAE53429
EtbAS	*Euphorbia tirucalli*	BAE43642
BkbAS	*Bupleurum kaoi*	AAS83468
AabAS	*Artemisia annua*	ACA13386
AebAS	*Aralia elata*	HM219225
SlbAS	*Solanum lycopersicum*	NP _001234604
OeLUS	*Olea europaea*	BAA86930
SlLUS	*Solanum lycopersicum*	NP _001352998
LjLUS	*Lotus japonicus*	BAE53430
ToLUS	*Taraxacum officinale*	BAA86932
SlCAS	*Solanum lycopersicum*	NP _ 001233784
AtCAS	*Arabidopsis thaliana*	AAC04931
AiCAS	*Azadirachta indica*	AGC82085
CaCAS	*Centella asiatica*	AAS01524
PnCAS	*Panax notoginseng*	ABY60426
RsCAS	*Rhizophora stylosa*	AB292608
PsCAS	*Pisum sativum*	D89619
PgOSC	*Panax ginseng*	AB009031
PgCAS	*Panax ginseng*	AB009029
PgDAS	*Panax ginseng*	AB265170
SiDAS	*Sesamum indicum*	XP 011096562
EgDAS	*Erythranthe guttata*	XP 012842229
CaDAS	*Coffea arabica*	XP 027088014
CeDAS	*Coffea eugenioides*	XP 027185776
